# Chemotherapy-induced peripheral neuropathy in breast cancer patients treated with eribulin: interim data from a post-marketing observational study

**DOI:** 10.1007/s12282-018-0919-8

**Published:** 2018-10-15

**Authors:** Junji Tsurutani, Yukinori Sakata, Toshiyuki Matsuoka

**Affiliations:** 1 Department of Medical Oncology, Kindai University Faculty of Medicine, 377-2, Onohigashi, Osaka-Sayama, Osaka 589-8511 Japan; 2 Advanced Cancer Translational Research Institute, Showa University, 1-5-8, Hatanodai, Shinagawa-ku, Tokyo, 142-8666 Japan; 3 Clinical Planning and Development Department, Eisai Co., Ltd., 4-6-10, Koishikawa, Bunkyo-ku, Tokyo, 112-8088 Japan

**Keywords:** Eribulin, Chemotherapy-induced peripheral neuropathy, Risk factors, Post-marketing study

## Abstract

**Background:**

Few studies have examined chemotherapy-induced peripheral neuropathy (CIPN) following the administration of eribulin as first- or second-line therapy in patients with breast cancer. We therefore assessed CIPN incidence by severity and risk factors for CIPN in patients treated with eribulin for HER2-negative inoperable or recurrent breast cancer, regardless of line therapy status.

**Methods:**

This multicenter, prospective, post-marketing observational study enrolled patients from September 2014 in Japan and followed them for 2 years. For this interim analysis, the data cut-off point was in November 2017. CIPN severity was assessed based on the Japanese version of the Common Terminology Criteria for Adverse Events, version 4.0.

**Results:**

Among 634 patients included in the safety analysis, 374 patients did not have existing CIPN at baseline. CIPN was observed in 105 patients (28.1%), including 67 (17.9%), 34 (9.1%), and 4 (1.1%) patients with grade 1, 2, and 3 severity, respectively. Of the 105 patients, 85.7% patients continued, 7.6% reduced, interrupted or postponed, and 6.7% discontinued eribulin. The median time (min‒max) from baseline to CIPN onset was 60 (3‒337) days. Multivariate logistic regression identified a significant association between CIPN and hemoglobin level at baseline, starting dose of eribulin, and history of radiotherapy.

**Conclusions:**

Our findings indicate that, with respect to CIPN, eribulin is well-tolerated, as approximately one-quarter of patients developed CIPN, most cases were grade 1 or 2, and the majority of patients continued eribulin after CIPN onset.

**Electronic supplementary material:**

The online version of this article (10.1007/s12282-018-0919-8) contains supplementary material, which is available to authorized users.

## Introduction

Chemotherapy-induced peripheral neuropathy (CIPN) is a common dose-limiting side effect of anticancer chemotherapeutic agents such as antitubulins (e.g., taxanes) and platinum analogs [1]. CIPN is primarily characterized by sensory and motor symptoms (e.g., numbness, tingling, pain in the extremities, and weakness) [2, 3]. A meta-analysis revealed that 30% of cancer patients continued to suffer from CIPN 6 months after chemotherapy, although the prevalence of CIPN decreased over time (68.1%, 60.0%, and 30.0% at 1 month, 3 months, and 6 months or more after chemotherapy, respectively) [4]. Given the progressive nature of CIPN, the persistence of its symptoms even months or years after the completion of chemotherapy [5], and its detrimental effects on the quality of life of cancer patients [6, 7], the treatment of CIPN may require dose reduction or cessation of chemotherapy, which may hamper treatment effectiveness and influence overall survival [1, 8, 9]. Previous studies have identified several risk factors for CIPN, including obesity [10], diabetes mellitus [11, 12], anemia [13], and reduced creatinine clearance [14]. To date, the pathophysiology of CIPN is not fully understood, and its effective management remains challenging [3, 4], thus highlighting the need to determine the actual situation of CIPN (e.g., CIPN incidence by severity and risk factors) in clinical settings to facilitate the development of preventive and therapeutic strategies.

Eribulin mesylate (Halaven^®^, Eisai Co., Ltd., Tokyo, Japan) is a nontaxane microtubule dynamics inhibitor, which was approved for the treatment of inoperable or recurrent breast cancer in Japan in 2011. Owing to its unique mechanism of action, which is unlike that of other chemotherapeutic agents such as paclitaxel and docetaxel, eribulin exerts antitumor activity and can prolong overall survival in patients with recurrent or metastatic breast cancer with well-defined taxane resistance [15–18]. However, the underlying mechanism by which eribulin is associated with the development of CIPN remains unclear, given its relatively low incidence reported in the literature [18–20].

Few studies on CIPN and eribulin in patients with breast cancer in clinical settings have been reported. Furthermore, data on CIPN in patients receiving eribulin as first- or second-line therapy can be collected only in Japan, as eribulin is approved in the United States as third- or later-line therapy and in Europe as second- and later-line therapy. We therefore conducted a 2-year post-marketing observational study to mainly assess CIPN incidence by severity and risk factors following eribulin treatment in patients with HER2-negative inoperable or recurrent breast cancer, regardless of the patient’s line therapy status. Here, we report data from the interim analysis of this study.

## Patients and methods

### Study design

This was a multicenter, prospective, post-marketing observational study conducted in Japan (ClinicalTrials.gov: NCT02371174). Patients were enrolled from September 2014 and followed for 2 years. The last enrollment was in February 2016 and the last follow-up visit was in February 2018.

For this interim analysis, the data cut-off point was 14th November 2017, and all data collected prior to this cut-off point were included. The final analysis is expected to be performed in 2019.

Eisai Co., Ltd. reviewed the scientific and ethical validity of the study design. This study was conducted in compliance with the Declaration of Helsinki and Japanese Good Post-Marketing Study Practice (GPSP), an authorized standard for post-marketing surveillance. In GPSP, approval is not required from either each institution’s institutional review board (IRB) and informed consent is not required from the participating patients. However, in practice, IRB approval or informed consent may be obtained if judged necessary by the institution. Personal data related to this study were handled in accordance with privacy protection laws in Japan.

### Patients

Eribulin-naïve patients with HER2-negative inoperable or recurrent breast cancer receiving eribulin as first-/second-line or as third-/later-line chemotherapy were recruited in approximately equal numbers. Pre- and post-operative chemotherapy, hormone therapy, antibody therapy, immunotherapy, and local radiation therapy were not included as previous regimens. HER2 status was defined according to the American Society of Clinical Oncology/College of American Pathologists HER2 Testing Guideline Update [21]. HER2-negative status was established if any of the following were applicable: (1) immunohistochemistry (IHC) 0 or 1 +; (2) IHC 2 + and negative gene amplification by fluorescence in situ hybridization (FISH); or (3) negative gene amplification by FISH. For analysis by FISH, HER2-negative status was defined as a HER2/CEP17 ratio < 2.0. The use of other gene amplification tests was also allowed in this study. Patients with severe bone-marrow suppression defined as a neutrophil count of < 1000/mm^3^ or platelet count < 75,000/mm^3^, patients with a history of hypersensitivity to the components of eribulin, or pregnant or possibly pregnant patients were excluded.

### Eribulin administration

Eribulin was generally administered intravenously at a dose of 1.4 mg/m^2^ over 2 to 5 min on day 1 (starting of eribulin treatment; baseline) and day 8 of a 21-day cycle as indicated. A lower starting dose (1.1 mg/m^2^) was recommended for some patients, such as those with hepatic dysfunction. Dosing was reduced depending on the patient’s condition to manage toxicity.

### Data collection

Patients were registered by central registration. Patient data were collected via registration forms and case report forms (CRFs). CRFs were collected after the following observation periods: (1) baseline to 6 months, (2) > 6 months after baseline to 1 year, and (3) > 1 year after baseline to 2 years. Patient outcomes (alive/dead) for patients who discontinued eribulin treatment before 2 years were collected until the end of the 2-year period from the first eribulin administration date. For patients who newly developed or worsened CIPN during eribulin treatment and did not recover from CIPN at the time of eribulin completion, CIPN data (e.g., outcome and drug used for treatment) were collected until patient recovery for a maximum of 2 years from the first eribulin administration date. No chemotherapy restrictions were set after the completion of eribulin treatment. Patients with treatment duration of ≥ 2 years were followed until the end of the 2-year period from the first eribulin administration date. Patients who could not be followed because they had died or were transferred to a different hospital within 2 years of the first eribulin administration date were followed for as long as possible.

### Assessment

Baseline characteristics (e.g., age, gender, and body weight), treatment history (e.g., operative treatment, pre- and post-operative chemotherapy, and chemotherapy for inoperable/recurrent breast cancer), eribulin administration (e.g., administered date and dose), eribulin treatment status (i.e., treatment continued/discontinued, reason for discontinuation), patient outcome (alive/dead), treatment for CIPN prevention and management, combination therapy (e.g., antineoplastic agents, hormone therapy, and radiotherapy), combination chemotherapy, chemotherapy used after eribulin treatment, and laboratory test results were investigated. CIPN data including severity, date of onset or worsening, outcome, and outcome date were assessed.

### Definition

CIPN severity was assessed based on the Japanese version of the Common Terminology Criteria for Adverse Events (CTCAE), version 4.0. For patients without existing CIPN at baseline, CIPN was defined as new onset of CIPN (grade ≥ 1) after eribulin administration. For patients with existing CIPN at baseline, CIPN was defined as worsening of CIPN (grade ≥ 1) after eribulin administration. A return in CTCAE grade to the baseline value was defined as recovery from CIPN. Improvement in CIPN was defined as a CTCAE grade that did not return to the baseline value but showed a ≤ 1 grade improvement from the worst grade with the investigator’s subsequent judgment of “improved”.

### Statistical analysis

For patients without existing CIPN at baseline, the data are summarized in the body of the text. For patients with existing CIPN at baseline, the following data are available as Supplementary Materials: CIPN incidence by severity grade, time to CIPN, outcome after CIPN, and time to recovery from or improvement of CIPN.

Baseline characteristics, CIPN incidence according to severity grade, CIPN incidence according to cumulative eribulin dose, eribulin treatment status after CIPN, time to CIPN, outcome after CIPN, and time to recovery or improvement from CIPN (the period from the development or worsening of CIPN to the recovery or improvement from CIPN during eribulin treatment) were summarized descriptively. For the patients who experienced CIPN during the study period, the time to CIPN and time to recovery or improvement from CIPN were calculated as median days (min–max). To assess the association between a given risk factor and CIPN onset, we conducted univariate and multivariate logistic regression analyses for each factor. First, odds ratio (OR) and 95% confidence interval were calculated for each factor, before a stepwise method was used with selection criteria of *p* < 0.20. All statistical factors with *p* < 0.05 were interpreted as statistically significant. All statistical analyses were performed using SAS software version 9.4 (SAS Institute, Inc., Cary, North Carolina).

## Results

### Patients

Of 651 patients registered from 183 institutions in this study, 634 patients were included in the safety analysis. Of those, 374 patients did not have existing CIPN, 257 patients had existing CIPN, and 3 patients were of unknown CIPN status at baseline. Baseline characteristics of the patients without CIPN at baseline (*n* = 374) are summarized in Table 1. The mean ± standard deviation (SD) age of patients was 58.4 ± 11.3 years, and all the patients were female. Overall, 8.0% of patients reported a history of CIPN from previous chemotherapy. Eribulin was started at 1.4 mg/m^2^ and reduced dose for 295 patients (78.9%) and 79 patients (21.1%), respectively. The reasons for starting at the reduced dose were mainly due to the deteriorated pathophysiological condition of the patients at baseline, such as advanced age and hepatic dysfunction. The mean (± SD) duration of patient observation was 410.9 ± 242.8 days (n = 372).

**Table 1:** Baseline characteristics

	*n* = 374
Gender, *n* (%)	
Female	374 (100)
Age (years)	
Mean ± SD	58.4 ± 11.3
Range	32–83
BMI (kg/m^2^), *n* (%)	
< 25	301 (80.5)
≥ 25	73 (19.5)
Starting dose of eribulin (mg/m^2^), *n* (%)	
≤ 1.1	64 (17.1)
> 1.1–1.4	310 (82.9)
Menopause status, *n* (%)	
Pre	62 (16.6)
Post	290 (77.5)
Unknown	22 (5.9)
Hormone receptor status, *n* (%)	
Positive	269 (71.9)
Negative	94 (25.1)
Unknown	11 (2.9)
Metastases, *n* (%)	
Bone	207 (55.3)
Liver	163 (43.6)
Lung	150 (40.1)
Distal lymph node	104 (27.8)
Regional lymph node	99 (26.5)
Skin	44 (11.8)
Brain	28 (7.5)
Affected side of breast	20 (5.3)
Healthy side of breast	9 (2.4)
Others	87 (23.3)
ECOG PS, *n* (%)	
0	233 (62.3)
1	123 (32.9)
2	14 (3.7)
3	4 (1.1)
History of radiotherapy, *n* (%)	
No	284 (75.9)
Yes	88 (23.5)
Unknown	2 (0.5)
Number of previous chemotherapy regimens, *n* (%)^a^	
0	118 (31.6)
1	104 (27.8)
2	78 (20.9)
3	29 (7.8)
4	33 (8.8)
≥ 5	12 (3.2)
Number of previous chemotherapy regimens, *n* (%)^a^	
≤ 1	222 (59.4)
≥ 2	152 (40.6)
History of taxane-based chemotherapy, n (%)	145 (38.8)
History of platinum-based chemotherapy, *n* (%)	1 (0.3)
CIPN history from previous chemotherapy, *n* (%)^b^	
No	330 (88.2)
Yes	30 (8.0)
Unknown	14 (3.7)
Complication of diabetes, *n* (%)	
No	351 (93.9)
Yes	23 (6.1)
Complication of liver dysfunction, *n* (%)	
No	342 (91.4)
Yes	32 (8.6)
Complication of hypertension, *n* (%)	
No	330 (88.2)
Yes	44 (11.8)
Hemoglobin at baseline (g/dL), *n* (%)	
< 11.5	136 (36.4)
≥ 11.5	233 (62.3)
AST at baseline (IU/L), *n* (%)	
< 32	213 (57.0)
≥ 32	145 (38.8)
Creatinine at baseline (mg/dL), *n* (%)	
< 0.7	277 (74.1)
≥ 0.7	79 (21.1)

*AST* aspartate transaminase, *BMI* body mass index, *ECOG PS* Eastern Cooperative Oncology Group Performance Status, *CIPN* chemotherapy-induced peripheral neuropathy, *SD* standard deviation

^a^Pre- and post-operative chemotherapy regimens were not included

^b^Response was categorized as “Yes” or “No” if the patient had or had not, respectively, experienced CIPN from any previous chemotherapy

### CIPN incidence stratified by CIPN severity, cumulative eribulin dose, and eribulin treatment status

**Fig. 1:**
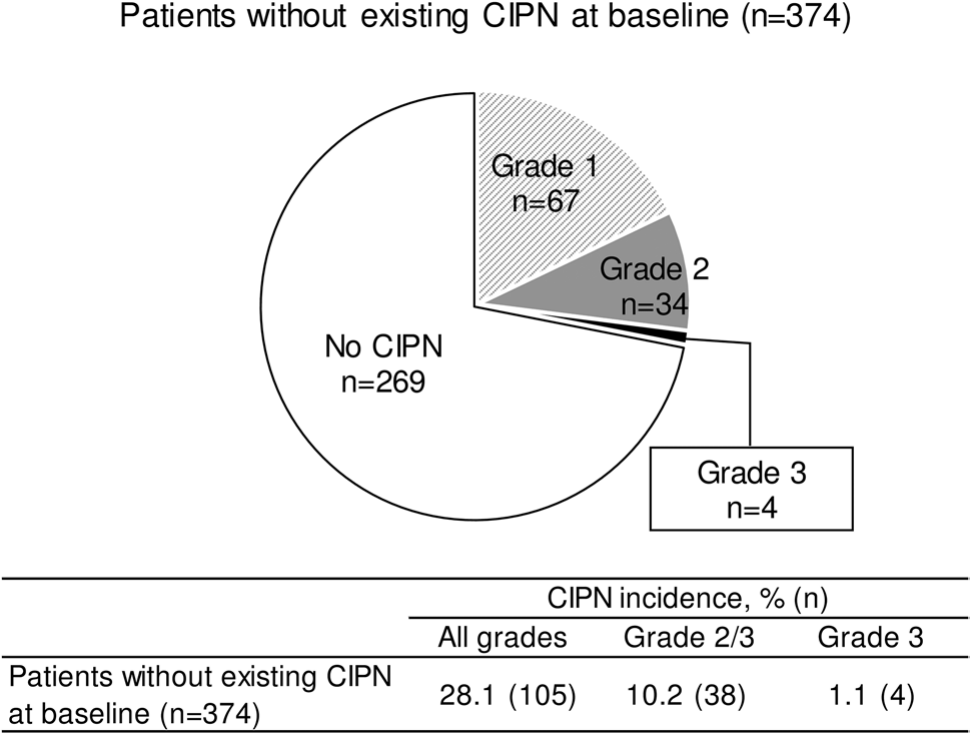
CIPN incidence by severity after eribulin treatment. *CIPN* chemotherapy-induced peripheral neuropathy

Among 374 patients without existing CIPN at baseline, CIPNs were observed in 105 patients (28.1%), including 67 patients (17.9%), 34 patients (9.1%), 4 patients (1.1%) at grade 1, 2, and 3, respectively (Fig. 1). CIPN incidence stratified by severity among the patients with existing CIPN at baseline is shown in Supplementary Fig. 1.

CIPN incidence stratified by the cumulative eribulin dose is summarized in Table 2. CIPN incidence was highest (51.1%) among the patients with the highest cumulative eribulin dose (> 22.4 mg/m^2^).

**Table 2:** CIPN incidence stratified by cumulative dose of eribulin

	*n* = 374	CIPN incidence	
		*n*	%
		105	28.1
Cumulative dose of eribulin (mg/m^2^)			
≤ 5.6	78	5	6.4
> 5.6–11.2	84	15	17.9
> 11.2–16.8	72	24	33.3
> 16.8–22.4	50	15	30.0
> 22.4	90	46	51.1

Of the 105 patients who developed new-onset CIPN, 7.6% (*n* = 8) were managed by a reduction, interruption, or postponement of eribulin treatment; 6.7% (*n* = 7) discontinued eribulin, and 85.7% (*n* = 90) continued on eribulin. Of those 105 patients, 44.8% (*n* = 47) received pharmacological treatment for CIPN, such as pregabalin, goshajinkigan, or mecobalamin.

### Time to CIPN onset

The time to CIPN onset is summarized in Table 3. The median (min‒max) time was 60 (3–337) days. Supplementary Table 1 shows the time to worsening CIPN for the patients with existing CIPN at baseline.

**Table 3:** Time to CIPN stratified by severity

	All grades		Grade 2/3		Grade 3	
	Median days (min‒max)	*n*	Median days (min‒max)	*n*	Median days (min‒max)	*n*
Patients without existing CIPN at baseline (*n* = 105)	60 (3‒337)	105	92 (8‒169)	21	127 (127‒127)	1

*CIPN* chemotherapy-induced peripheral neuropathy

### Outcome after CIPN and time to recovery or improvement from CIPN

Of 105 patients who had developed CIPN from baseline, 56.2% (*n* = 59) recovered or improved from CIPN. Median time (min‒max) to recovery or improvement from CIPN was 134.0 (5‒760) days. Supplementary Table 2 shows CIPN outcome for the patients with existing CIPN at baseline.

### Risk factors for CIPN onset

The results of univariate and multivariate logistic regression analyses are shown in Table 4. Univariate logistic regression analysis showed that BMI, starting dose of eribulin, history of radiotherapy, and hemoglobin level at baseline were significantly associated with CIPN onset. Multivariate logistic regression analysis showed that hemoglobin level at baseline (OR = 2.415, *P* = 0.004), starting dose of eribulin (OR = 2.748, *P* = 0.026), and history of radiotherapy (OR = 0.366, *P* = 0.008) were significantly associated with CIPN onset. CIPN history from previous chemotherapy was not significantly associated with CIPN. No association was found for the number of previous chemotherapy regimens.

**Table 4:** Univariate and multivariate logistic regression analyses of associations between CIPN and risk factors

Factor	Category	Univariate logistic regression					Multivariate logistic regression				
		n	Patients who developed CIPN, *n*	OR	(95% CI)	*P* value	n	Patients who developed CIPN, *n*	OR	(95% CI)	*P* value
Age (years)	< 65	234	66				192	50			
	≥ 65	140	39	0.983	(0.616, 1.567)	0.942	121	32			
BMI (kg/m^2^)	< 25	301	77				258	63			
	≥ 25	73	28	1.810	(1.057, 3.101)	0.031	55	19	1.654	(0.846, 3.234)	0.141
Starting dose of eribulin (mg/m^2^)	≤ 1.1	64	10				54	7			
	> 1.1–1.4	310	95	2.386	(1.165, 4.886)	0.017	259	75	2.748	(1.130, 6.685)	0.026
Menopause	Pre	62	15				58	13			
	Post	290	82	1.235	(0.655, 2.331)	0.514	255	69			
ECOG PS	0	233	72				197	56			
	≥ 1	141	33	0.683	(0.423, 1.103)	0.119	116	26			
History of radiotherapy	No	284	93				241	72			
	Yes	88	12	0.324	(0.168, 0.626)	0.001	72	10	0.366	(0.173, 0.772)	0.008
History of hormone therapy	No	109	26				91	20			
	Yes	265	79	1.356	(0.812, 2.265)	0.245	222	62			
Number of previous chemotherapy regimens^a^	≤ 1	222	64				188	52			
	≥ 2	152	41	0.912	(0.575, 1.446)	0.695	125	30			
CIPN history from previous chemotherapy^b^	No	330	88				284	70			
	Yes	30	12	1.834	(0.849, 3.961)	0.123	29	12	2.174	(0.932, 5.074)	0.072
Lung metastasis	No	224	64				189	52			
	Yes	150	41	0.940	(0.593, 1.492)	0.794	124	30			
Liver metastasis	No	211	57				175	43			
	Yes	163	48	1.128	(0.716, 1.775)	0.604	138	39			
Bone metastasis	No	167	49				135	35			
	Yes	207	56	0.893	(0.568, 1.405)	0.625	178	47			
Complication of diabetes	No	351	96				296	76			
	Yes	23	9	1.708	(0.716, 4.075)	0.228	17	6			
Complication of liver dysfunction	No	342	97				283	75			
	Yes	32	8	0.842	(0.366, 1.939)	0.686	30	7			
Complication of hypertension	No	330	94				276	73			
	Yes	44	11	0.837	(0.406, 1.725)	0.629	37	9			
Drug for CIPN prevention	No	342	92				283	70			
	Yes	32	13	1.860	(0.883, 3.917)	0.103	30	12	2.307	(0.998, 5.335)	0.051
Hemoglobin at baseline (g/dL)	< 11.5	136	23				117	20			
	≥ 11.5	233	82	2.668	(1.582, 4.500)	0.000	196	62	2.415	(1.329, 4.389)	0.004
AST at baseline (IU/L)	< 32	213	63				184	49			
	≥ 32	145	37	0.816	(0.507, 1.312)	0.401	129	33			
Creatinine at baseline (mg/dL)	< 0.7	277	79				239	64			
	≥ 0.7	79	20	0.850	(0.480, 1.503)	0.575	74	18			

*AST* aspartate transaminase, *BMI* body mass index, *CI* confidence interval, *CIPN* chemotherapy-induced peripheral neuropathy, *ECOG PS* Eastern Cooperative Oncology Group Performance Status, *OR* odds ratio

^a^Pre- and post-operative chemotherapy regimens were not included

^b^Response was categorized as “Yes” or “No” if the patient had or had not, respectively, experienced CIPN from any previous chemotherapy

## Discussion

Given the lack of data on CIPN associated with eribulin as first- or second-line therapy in patients with breast cancer, we conducted a 2-year post-marketing observational study to assess CIPN incidence by severity and the risk factors for CIPN in patients with HER2-negative inoperable or recurrent breast cancer treated with eribulin, regardless of the patient’s line therapy status. The overall CIPN incidence was 28.1%, and most cases were grade 1 or 2, with the majority of patients continuing eribulin treatment after CIPN onset. Furthermore, more than 50% of patients who developed CIPN recovered or improved within 6 months. Multivariate logistic regression analysis showed that hemoglobin level at baseline, starting dose of eribulin, and history of radiotherapy were significantly associated with CIPN onset.

CIPN incidence observed in this study was approximately 10% higher than that reported in a previous 1-year post-marketing study of eribulin in Japan (16.8%), although no notable difference in the incidence of high-grade CIPN was found (grade ≥ 3 CIPN: 1.1% and 2.7% in this study and the previous study, respectively) [20]. The reason for the higher CIPN incidence observed in this study is unclear, although it may be attributable to the longer study duration of this study. Additionally, because the study outcome specifically focused on CIPN, increased reporting of CIPN as an AE may have contributed to a possible overestimation of CIPN incidence. When compared with CIPN incidence in a meta-analysis of eribulin, similar results were noted (CIPN incidence in the meta-analysis: 27.5%; high-grade CIPN incidence: 4.7%) [22]. Given that CIPN incidence in this study included patients receiving eribulin as first- or second-line therapy were consistent with previous studies, that most cases of CIPN were grade 1 or 2, and that most patients continued eribulin treatment after CIPN onset, our findings indicate that eribulin is well-tolerated with respect to CIPN, regardless of the patient’s line therapy status.

Taxane-based chemotherapy such as paclitaxel is reported to induce a relatively high CIPN (58.4‒73.0% of patients receiving taxane-based chemotherapy [4, 10, 23, 24]). However, direct comparison with previous studies is difficult because of differences in study design (e.g., CIPN assessment and study duration), and CIPN incidence in the present study was lower than that observed following taxane-based chemotherapy. Furthermore, in accordance with previous studies, CIPN incidence following treatment with eribulin was relatively low [18–20]. Preclinical studies have shown that this low incidence might be attributable to differences in the mechanism of action between eribulin and other chemotherapeutic agents (e.g., paclitaxel) [25–28], although the underlying mechanism by which CIPN is associated with eribulin requires further exploration. Thus, considering the relatively low CIPN incidence observed following treatment with eribulin, eribulin may represent a more tolerable treatment option for patients with potentially predisposing factors for CIPN (e.g., obesity and diabetes mellitus).

Time to CIPN onset in the present study was notably longer than that reported for taxane-based chemotherapy (paclitaxel and docetaxel), with an average duration of 3.8 weeks to the first occurrence of CIPN in a previous study of taxane-based chemotherapy study [23] compared with the 60 days observed in the present study. As described above, findings from preclinical studies have shown that CIPN may vary in terms of the initiation, progression, persistence, and recovery from CIPN by the distinct mechanistic actions of other chemotherapies (e.g, eribulin and paclitaxel) [25–28], indicating that the mechanism of action of eribulin might, at least partially, be responsible for the comparatively long time to CIPN onset observed in this study. Further studies on CIPN and eribulin are warranted to elucidate the mechanism of action of eribulin.

Risk factors for CIPN onset identified with statistical significance in this study were starting dose of eribulin (> 1.1–1.4 mg/m^2^), hemoglobin level at baseline (≥ 11.5 g/dL), and history of radiotherapy. Risk factors as the starting dose of eribulin (> 1.1–1.4 mg/m^2^) and BMI (≥ 25 kg/m^2^), which showed no statistical significance in this study, but are reported risk factors for CIPN among cancer patients [29, 30], may be explained by the dose of eribulin that patients receive. Given their higher body surface area [31], obese patients typically receive higher doses of chemotherapy than non-obese patients, which may result in a higher CIPN incidence. In this study, those factors might have contributed to CIPN onset among obese patients who received a higher starting dose of eribulin. In support of this possibility, we found that CIPN incidence was generally higher in patients who received higher cumulative eribulin doses, as shown in Table 2. Other reported risk factors for CIPN include baseline neuropathy, presence of diabetes, smoking history (pack-year), decreased creatinine clearance, specific sensory changes during chemotherapy, and age [4, 12, 14, 32, 33]. In this study, we found no significant associations between CIPN and several of these risk factors, namely, age, diabetes, and reduced creatinine clearance at baseline. Because the risk factors of CIPN vary depending on the type of chemotherapy, type of cancer, and race, further studies are warranted to more comprehensively identify the risk factors for CIPN induced by eribulin.

In a previous study in daily practice, eribulin starting dose was reduced in 23.5% of the patients who would have been ineligible for the phase III EMBRACE study [34]. Similarly in this study, 21.1% of the patients received the reduced starting dose owing to the safety concerns. The package insert in Japan suggests a starting dose of 1.4 mg/m^2^; a reduced dose of 1.1 mg/m^2^ is recommended for some patients (e.g., those with hepatic dysfunction). Hence, because some patients start eribulin at reduced dose depending on patients’ pathophysiological condition in clinical settings, the safety results might possibly differ between phase III trials and studies in practice, as demonstrated in a previous study [35]. Indeed, CIPN incidence in this study was lower than the phase III EMBRACE study (PN incidence 35% and 8% for all grades and grade 3/4, respectively) [15].

Some findings of this study should be interpreted with caution. First, the results could be due to chance owing to the relatively small number of patients included in the analyses. Second, CIPN incidence may have been underestimated, as some patients discontinued eribulin early or died following disease progression before CIPN onset was observed. Third, patients who underwent radiotherapy within the 6 months prior to eribulin administration (baseline) were considered to have a history of radiotherapy. Thus, the effect on patients who underwent radiotherapy before the 6 months prior to baseline cannot be determined. Finally, among the various mechanisms available to assess CIPN severity, the Japanese version of the CTCAE version 4.0 was used, but other methods might have produced different results.

In conclusion, our findings suggest that eribulin is well-tolerated with respect to CIPN onset, as approximately one-quarter of patients experienced CIPN, most cases of which were grade 1 or 2, and most patients continued to receive eribulin after CIPN onset.

## Electronic supplementary material

Below is the link to the electronic supplementary material.

Supplementary material 1 (RTF 10599 KB)
