# Supplementary material for: Chemotherapy-induced peripheral neuropathy in breast cancer patients treated with eribulin: interim data from a post-marketing observational study
Source: Breast Cancer. 2018 Oct 15;26(2):235–43. doi: 10.1007/s12282-018-0919-8 (PMC6394617; doi:10.1007/s12282-018-0919-8)
Supplement: Supplementary file 1 — Supplementary material 1 (RTF 10599 KB) [file 12282_2018_919_MOESM1_ESM.rtf]

Supplementary Materials
Supplementary Figure 1. CIPN incidence by severity after eribulin treatment among the patients with existing CIPN at baseline 

a) For patients with existing CIPN at baseline, CIPN were defined as an AE where CIPN at baseline subsequently worsened.


Supplementary Table 1. Time to CIPN stratified by severity among the patients with existing CIPN at baseline
	All grades	Grade 2/3 	Grade 3 	
	Median days (min‒max)	n	Median days (min‒max)	n	Median days (min‒max)	n	
Patients with existing CIPN at baseline (n=59)	71
(1‒386)	59	73
(1‒339)	49	101
(23‒117)	5	
CIPN chemotherapy-induced peripheral neuropathy


Supplementary Table 2. Outcome after CIPN and time to recovery or improvement from CIPN among the patients with existing CIPN at baseline
	Patients who recovered or improved from CIPN	Time to recovery or improvement from CIPN	
	%, (n)	Median days, (min–max)	
Patients with existing CIPN at baseline (n=59)	50.8 (30)	134.0	(8–569)	
CIPN chemotherapy-induced peripheral neuropathy
